# Heyde-like syndrome occurring for the first time following aortic valve replacement with a bioprosthesis: a case report

**DOI:** 10.1186/s44215-023-00066-x

**Published:** 2023-09-04

**Authors:** Takeshi Oda, Ryo Kanamoto, Mizue Miyawaki, Keiichi Akaiwa, Katsuhiko Nakamura, Minako Kubochi, Seiya Kato, Eiki Tayama

**Affiliations:** 1 Division of Cardiovascular Surgery, Omura Municipal Hospital, Omura City, Nagasaki, 856-8561 Japan; 2 Division of Internal Medicine, Omura Municipal Hospital, Omura City, Nagasaki, Japan; 3 Division of Pathology, Saiseikai Fukuoka General Hospital, Fukuoka, Japan; 4 Department of Cardiovascular Surgery, Kurume University School of Medicine, Kurume City, Fukuoka, Japan

**Keywords:** Heyde’s syndrome, Angiodysplasia, Structural valve deterioration, Aortic valve stenosis, Aortic valve replacement

## Abstract

**Background:**

Heyde’s syndrome is known as a combination of gastrointestinal (GI) bleeding and aortic valve stenosis. However, there are no reports of an association between GI bleeding and bioprosthetic valve stenosis initially occurred after aortic valve replacement (AVR), even though there are several reports that GI bleeding due to native aortic valve stenosis disappeared after AVR, and GI bleeding recurred due to bioprosthetic valve stenosis or patient-prosthesis mismatch.

**Case presentation:**

An 80-year-old woman who was on hemodialysis for 13 years had undergone AVR with a bioprosthetic valve for aortic regurgitation 3 years prior. She was admitted with acute heart failure and anemia that required repeated blood transfusions. Capsule endoscopy revealed multiple active hemorrhages of the small intestine due to angiodysplasia. Echocardiography showed severe bioprosthetic valve stenosis in the aortic valve position as a result of structural valve deterioration (SVD). Because Heyde’s syndrome was strongly suspected even though gel electrophoresis analysis of von Willebrand factor multimers, the gold standard examination for the definitive diagnosis of Heyde’s syndrome, was not performed, a redo AVR with a new bioprosthetic valve was performed. After the second AVR, both the heart failure and anemia due to GI bleeding promptly improved.

**Conclusions:**

Bioprosthetic valve stenosis due to SVD can bring GI bleeding just as in native aortic valve stenosis. Redo AVR is a promising treatment if the combination of GI bleeding and valve stenosis in the aortic valve position appears even after AVR.

## Background

The co-occurrence of gastrointestinal (GI) bleeding and aortic valve stenosis (AS) is known as Heyde’s syndrome [1]. In patients with Heyde’s syndrome, aortic valve replacement (AVR) often ceases GI bleeding [2–4], and bioprosthetic valve is recommended to avoid prolonged anticoagulant therapy [2, 3]. Herein, we illustrate a woman on hemodialysis (HD) with GI bleeding and hemodynamic AS that developed 3 years after AVR with a bovine pericardial bioprosthetic valve.

## Case presentation

An 80-year-old woman, who had a 13-year history of HD for end-stage kidney disease, had undergone AVR with a 21-mm Carpentier-Edwards Perimount Magna bioprosthesis (Edwards Lifesciences, Irvine, CA, USA) for severe aortic regurgitation 3 years earlier.

**Fig. 1:**
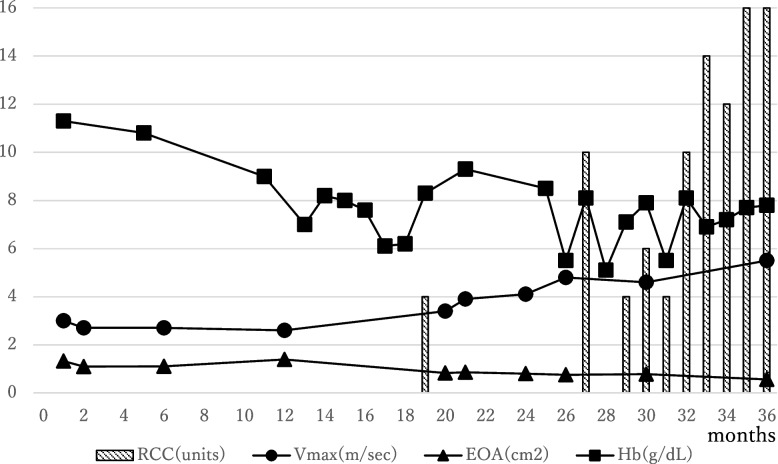
Transition of amount of transfusion of RCC, Vmax, MPG, and EOA after the initial AVR. RCC, red cell concentrate; Vmax, maximum velocity; MPG, mean pressure gradient; EOA, effective orifice area; AVR, aortic valve replacement

She recovered uneventfully. However, follow-up transthoracic echocardiography started to reveal hemodynamic AS due to structural deterioration (SVD) 20 months after the AVR. The severity of hemodynamic AS worsened, with a peak velocity of 5.5 m/s at the aortic valve position, a mean pressure gradient of 67 mmHg, and an effective aortic prosthetic valvular orifice area of 0.56 cm^2^ 3 years after AVR (Fig. 1).

**Fig. 2:**
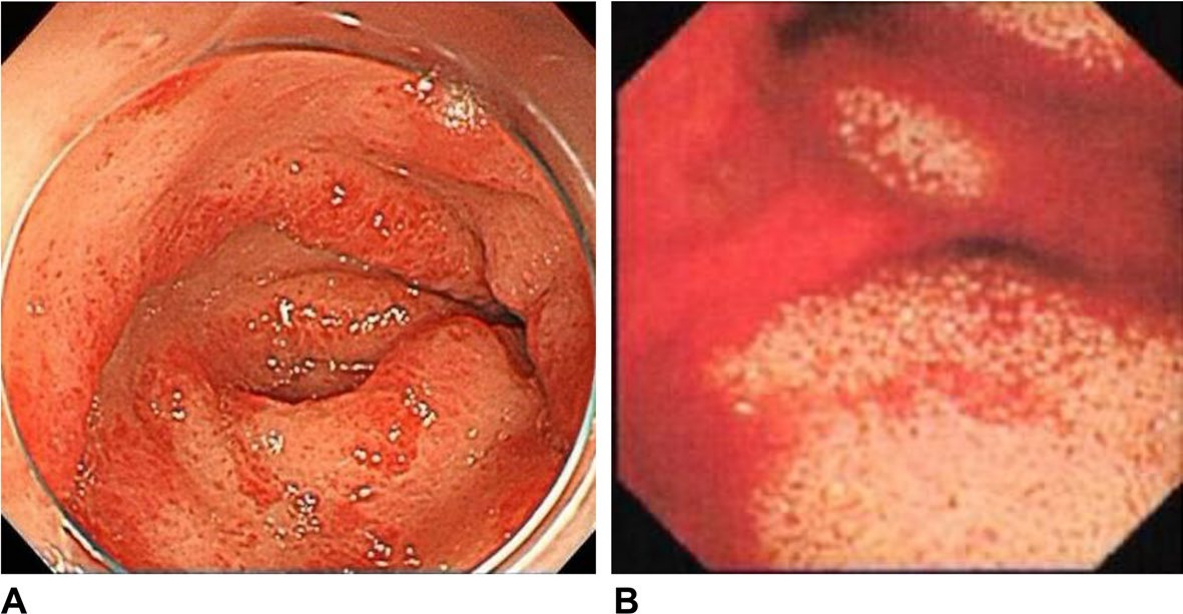
**A** Esophagogastroduodenoscopy revealing gastric telangiectasia and oozing of the gastric antrum. **B** Capsule endoscopy showing multiple active bleeding in the small intestine

From 27 months after AVR, she received repetitive blood transfusions for refractory anemia. Esophagogastroduodenoscopy revealed gastric telangiectasia and oozing hemorrhage in the gastric antrum (Fig. 2A). Colonoscopy did not reveal any bleeding. Although repeated endoscopic laser coagulation for hemostasis of the stomach was temporarily effective, bowel bleeding recurred. Capsule endoscopy showed multiple active bleeding in the small intestine (Fig. 2B).

She then started complaining of increased dyspnea and easy fatigability. Hypotension and frequent premature ventricular contractions were also observed during HD.

**Fig. 3:**
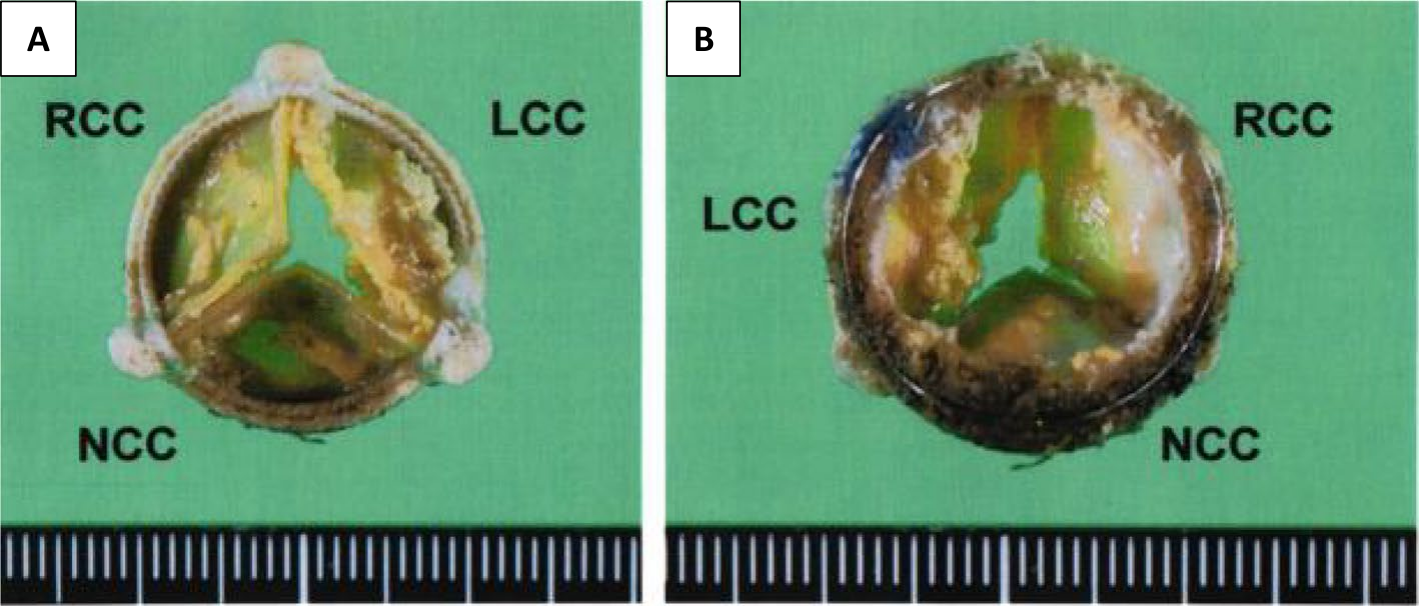
**A** Aortic aspect of the extracted Carpentier-Edwards Perimount Magna bioprosthetic valve. **B** Left ventricular aspect. The extracted bioprosthetic valve shows mineralization of all the leaflets and loss of mobility of the leaflet in the direction of the left coronary cusp. LCC, left coronary cusp; RCC, right coronary cusp; NCC, noncoronary cusp

Heyde’s syndrome was strongly suspected, although hemodynamic AS occurred not in the native aortic valve but in the implanted bovine pericardial bioprosthetic valve. Platelet counts, activated partial thromboplastin time, and prothrombin time-international normalized ratio were 5.7 × 10^4^ /μl, 34.7 s, and 1.06, respectively. The gel electrophoresis analysis of von Willebrand factor (vWF) multimers was not performed. She underwent a redo AVR with a 19-mm Edwards Inspiris Resilia bioprosthesis (Edwards Lifesciences, Irvine, CA, USA) under standard cardiopulmonary bypass. All leaflets of the explanted bioprosthetic valve were calcified on both the aortic and left ventricular sides, and the leaflet in the direction of the left coronary cusp had lost its mobility (Fig. 3).

**Fig. 4:**
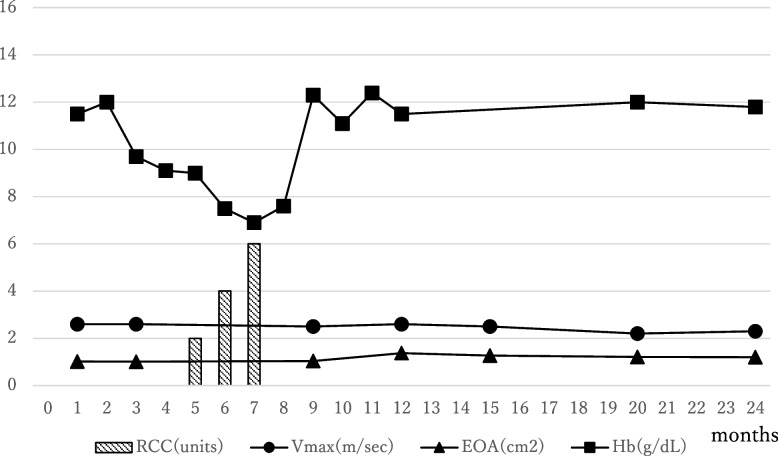
Transition of amount of transfusion of RCC, Vmax, MPG, and EOA after the redo AVR

After the second AVR, refractory anemia promptly improved. The disappearance of telangiectasia and oozing hemorrhage in the stomach were confirmed by esophagogastroduodenoscopy, which was performed 4 months after the second AVR. The hemoglobin level was stable without further episodes of GI bleeding even though small blood transfusions were required at 5, 6, and 7 months after the second AVR (Fig. 4). At that time, neither fecal occult blood nor findings of hemolytic anemia were seen; thus, the cause of the anemia was likely due to chronic kidney disease. The hemodynamic performance of the second implanted bioprosthetic valve in the aortic position has been satisfactory for 2 years since the redo AVR (Fig. 4).

## Discussion and conclusions

In 1958, Heyde reported an association between AS and GI bleeding [1]. Since then, several similar cases with the combination of AS and GI bleeding have been reported, which is clinically known as “Heyde’s syndrome” [2–4]. AS generates high blood flow through the stenotic aortic valve that leads to high shear stress and mechanical disruption of the high-molecular-weight (HMW) multimer of vWF.

The vWF is an essential factor in platelet aggregation. In particular, the HMW multimer of vWF is essential for its hemostatic function [5]. Type 2A von Willebrand disease occurs due to the lack of an HMW multimer. Therefore, AS can cause this coagulation disorder [6]. Heyde’s syndrome can be confirmed in cases of acquired type 2A von Willebrand disease. Vincentelli et al. reported a negative correlation between the percentage of HMW multimers and the mean transvalvular gradient through the aortic valve [7]. Therefore, the HMW multimer deficit is thought to be the cause of coagulopathy in Heyde’s syndrome. Meanwhile, Undas et al. reported a case of Heyde’s syndrome without a decrease in large vWF multimers and concluded that Heyde’s syndrome is not always associated with acquired von Willebrand syndrome [8]. Gel electrophoresis, the gold standard examination for definitive diagnosis of Heyde’s syndrome, was not performed in our patient because it is expensive, resource intensive [3], time-consuming, and is not covered by the public health insurance system in Japan.

A relationship with GI bleeding of unknown origin has been observed in 3% of severe AS cases [3]. Severe AS occurs in 15–25% of patients with repeated bleeding [9]. Cody et al. reported a greater than 100-fold increased incidence of idiopathic GI bleeding in AS cases compared to general hospital cases [10]. Typically, the GI bleeding source in AS is angiodysplasia in the small intestine or colon; therefore, a whole intestinal examination is necessary. Although repeated endoscopic cauterization was temporarily effective for gastric angiodysplasia in our patient, the GI bleeding recurred. Capsule endoscopy was useful for identifying bleeding lesions in the small intestine.

The effectiveness of AVR in stopping the GI bleeding has been reported in 80–93% of patients with Heyde’s syndrome [2, 11, 12]. The use of a bioprosthetic valve rather than a mechanical valve may be preferable to avoid the long-term anticoagulation requirement [3]. Thus, AVR with a bioprosthesis should be the first-line therapy for Heyde’s syndrome. Prosthetic valve selection for dialysis patients remains controversial. Since the life expectancy of these patients is poor, a bioprosthetic valve is generally recommended for elderly patients undergoing HD [13–15]. There are some reports indicating superior durability of bovine pericardial bioprostheses than that of porcine bioprostheses [16, 17]. However, there are no studies comparing the durability between bovine pericardial valve and porcine valve in HD patients. Our patient underwent the first AVR with a bovine pericardial bioprosthetic valve. Only 3 years later, the second AVR was required for Heyde-like syndrome. Although prosthetic valve selection was difficult, a bovine pericardial bioprosthetic valve was chosen because of her age and the existence of GI bleeding.

Some studies have reported transcatheter aortic valve implantation (TAVI) for patients with Heyde’s syndrome [18, 19]. However, TAVI for HD patients was approved in January 2021 and became available in Japan from February 2021. TAVI was not available during the time of second surgery for the patient. Soran et al. reported a patient with Heyde’s syndrome whose bleeding stopped immediately after AVR with a bioprosthetic valve and recurred when the implanted bioprosthetic valve was re-stenosed [20]. Vincentelli et al. reported a recurrence of hemostatic abnormalities in a case of a patient-prosthesis mismatch, even after AVR [7]. When a bioprosthetic valve is used for AVR, tissue valve stenosis due to SVD leads to coagulopathy recurrence and resolves again after redo AVR [21]. In our patient, coagulopathy did not occur prior to the initial AVR. This is probably because the first AVR was performed for aortic regurgitation rather than for AS. To the best of our knowledge, this is the first report on initial occurrence of Heyde-like syndrome following AVR.

Our patient recovered expeditiously after the second AVR and has remained well for the following 2 years. Redo AVR resulted in an immediate and sustainable cessation of bleeding and rapid resolution of chronic anemia. However, circumspect follow-up is mandatory.

In conclusion, the association between GI bleeding and valve stenosis in the aortic valve position can occur even after AVR because of bioprosthetic valve stenosis secondary to SVD. When Heyde’s syndrome is suspected even after AVR, redo AVR is a promising treatment.

## Data Availability

The data used and analyzed during the present study are available from the corresponding author on reasonable request.

## Abbreviations

AS: Aortic valve stenosis
AVR: Aortic valve replacement
EOA: Effective orifice area
GI: Gastrointestinal
HD: Hemodialysis
HMW: High molecular weight
MPG: Mean pressure gradient
RCC: Red cell concentrate
SVD: Structural valve deterioration
Vmax: Maximum velocity
vWF: Von Willebrand factor

## References

[1] Heyde EC. Gastrointestinal bleeding in aortic stenosis. N Engl J Med. 1958;259:196.

[2] Scheffer SM, Leatherman LL. Resolution of Heyde’s syndrome of aortic stenosis and gastrointestinal bleeding after aortic valve replacement. Ann Thorac Surg. 1986;42:477–80. 3490235 10.1016/s0003-4975(10)60563-2

[3] Pate GE, Chandavimol M, Naiman SC, Webb JG. Heyde’s syndrome: a review. J Heart Valve Dis. 2004;13:701–12. 15473466

[4] Islam S, Cevik C, Islam E, Attaya H, Nugent K. Heyde’s syndrome: a critical review of the literature. J Heart Valve Dis. 2011;20:366–75. 21863647

[5] Matsumoto M, Kawaguchi S, Ishizashi H, Yagi H, Iida J, Sasaki T, et al. Platelets treated with ticlopidine are less reactive to unusually large von Willebrand factor multimers than are those treated with aspirin under high shear stress. Pathophysiol Haemos Thromb. 2005;34:35–40. 10.1159/000088546 16293984

[6] Sadler JE. Aortic stenosis, von Willebrand factor, and bleeding. N Engl J Med. 2003;349:323–5. 12878737 10.1056/NEJMp030055

[7] Vincentelli A, Susen S, Le Tourneau T, Six I, Fabre O, Juthier F, et al. Acquired von Willebrand syndrome in aortic stenosis. N Engl J Med. 2003;349:343–9. 12878741 10.1056/NEJMoa022831

[8] Undas A, Windyga J, Bykowska K, Dimitrow PP, Stepien E, Sadowski J. Heyde’s syndrome without a decrease in large von Willebrand factor multimers: a case of intestinal bleeding reversed by valve replacement in a patient with aortic stenosis. Thromb Haemost. 2009;101:773–4. 19350125

[9] Yoshida K, Tobe S, Kawata M, Yamaguchi M. Acquired and reversible von Willebrand disease with high shear stress aortic valve stenosis. Ann Thorac Surg. 2006;81:490–4. 16427837 10.1016/j.athoracsur.2005.07.074

[10] Cody MC, O’Donovan TP, Hughes RW Jr. Idiopathic gastrointestinal bleeding and aortic stenosis. Am J Dig Dis. 1974;19:393–8. 4545225 10.1007/BF01255601

[11] King RM, Pluth JR, Giuliani ER. The association of gastrointestinal unexplained bleeding with calcific aortic stenosis. Ann Thorac Surg. 1987;44:514–6. 3499881 10.1016/s0003-4975(10)62112-1

[12] Thompson JL, Schaff HV, Dearani JA, Park SJ, Sundt TM III, Suri RM, et al. Risk of recurrent gastrointestinal bleeding after aortic valve replacement in patients with Heyde syndrome. J Thorac Cardiovasc Surg. 2012;144:112–6. 21864855 10.1016/j.jtcvs.2011.05.034

[13] Manghelli JL, Carter DI, Khiabani AJ, Gauthier JM, Moon MR, Munfakh NA, et al. A-20-year multicenter analysis of dialysis-dependent patients who had aortic or mitral valve replacement: implications for valve selection. J Thorac Cardiovasc Surg. 2019;158:805–13. 30685160 10.1016/j.jtcvs.2018.10.168 PMC6709586

[14] Nakatsu T, Minakata K, Tanaka S, Minatoya K. Intermediate-term outcomes of aortic valve replacement with bioprosthetic or mechanical valves in patients on hemodyalysis. J Thorac Cardiovasc Surg. 2019;157:2177–86. 31307139 10.1016/j.jtcvs.2018.08.104

[15] Okada N, Tajima K, Takami Y, Kato W, Fujii K, Hibino M, et al. Valve selection for the aortic position in dialysis patients. Ann Thorac Surg. 2015;99:1524–31. 25678501 10.1016/j.athoracsur.2014.11.055

[16] Webb J, Parkin D, Tondel K, Simitsis P, Roxburgh J, Chambers JB. A comparison of early redo surgery rates in mosaic porcine and perimount bovine pericardial valves. Eur J Cardiothorac Surg. 2018;54:724–8. 29579171 10.1093/ejcts/ezy113

[17] Persson M, Glaser N, Franco-Cereceda A, Nilsson J, Holzmann MJ, Sartipy U. Porcine vs bovine bioprosthetic aortic valves: long-term clinical results. Ann Thorac Surg. 2021;111:529–36. 32693042 10.1016/j.athoracsur.2020.05.126

[18] Godino C, Lauretta L, Pavon AG, Mangieri A, Viani G, Chieffo A, et al. Heyde’s syndrome incidence and outcome in patients undergoing transcatheter aortic valve implantation. J Am Coll Cardiol. 2013;61:687–9. 23391203 10.1016/j.jacc.2012.10.041

[19] Tsuchiya S, Matsumoto Y, Doman T, Fujiya T, Sugisawa J, Sato K, et al. Disappearance of angiodysplasia following transcatheter aortic valve implantation in a patient with Heyde’s syndrome: a case report and review of the literature. J Atheroscler Thromb. 2020;27:271–7. 31378751 10.5551/jat.49239 PMC7113142

[20] Soran H, Lewis M, Whorwell PJ. Bleeding angiodysplasia: should we concentrate more on the aortic valve than on the bowel? Int J Clin Pract. 2002;56:155–6. 11926706

[21] Abi-Akar R, El-Rassi I, Karam N, Jassar Y, Slim R, Jebara V. Treatment of Heyde’s syndrome by aortic valve replacement. Curr Cardiol Rev. 2011;7:47–9. 22294975 10.2174/157340311795677699 PMC3131716

